# Impact of Notch Signaling on Inflammatory Responses in Cardiovascular Disorders

**DOI:** 10.3390/ijms14046863

**Published:** 2013-03-26

**Authors:** Thibaut Quillard, Beatrice Charreau

**Affiliations:** 1 Division of Cardiovascular Medicine, Department of Medicine, Brigham and Women’s Hospital, Harvard Medical School, 77 Avenue Louis Pasteur, Boston, MA 02115, USA; E-Mail: tquillard@rics.bwh.harvard.edu; 2 INSERM U1064, Nantes F44000, France; 3 CHU Nantes, Institut de Transplantation et de Recherche en Transplantation Urologie Nephrologie, ITUN, Nantes F44000, France; 4 Université de Nantes, Faculté de Médecine, Nantes F44000, France

**Keywords:** cardiovascular diseases, endothelial cells, inflammation, Notch signaling, signaling pathways, vascular cells

## Abstract

Notch signaling is a major pathway in cell fate decisions. Since the first reports showing the major role of Notch in embryonic development, a considerable and still growing literature further highlights its key contributions in various pathological processes during adult life. In particular, Notch is now considered as a major player in vascular homeostasis through the control of key cellular functions. In parallel, confounding evidence emerged that inflammatory responses regulate Notch signaling *in vitro* in endothelial cells, smooth muscle cells or vascular infiltrating cells and *in vivo* in vascular and inflammatory disorders and in cardiovascular diseases. This review presents how inflammation influences Notch in vascular cells and, reciprocally, emphasizes the functional role of Notch on inflammatory processes, notably by regulating key cell functions (differentiation, proliferation, apoptosis/survival, activation). Understanding how the disparity of Notch receptors and ligands impacts on vasculature biology remains critical for the design of relevant and adequate therapeutic strategies targeting Notch in this major pathological context.

## 1. Introduction

Notch signaling is a major intercellular communication pathway highly conserved through evolution. Originally identified in *Drosophila*, in which a mutant allele gave rise to a notched wing [1], the Notch pathway was then reported to be a crucial regulator during embryonic development in vertebrate and in invertebrate organisms. Notch is also clearly implicated in many cellular processes, like differentiation, activation, apoptosis and proliferation in a wide array of cell types during adult life [2].

Notch receptors and ligands are type 1 transmembrane proteins. In mammals, four Notch receptors (Notch1-4) have been identified and five Notch ligands (Jagged [Jag]1 and Jag2 from the Jagged/serrate family and Dll1, three and four from the Delta-like family [Dll]). This generates a large number of receptor-ligand combinations, which could potentially generate distinct responses. However, whether particular receptor-ligand combinations provide selective Notch signaling is still largely unknown. In addition to the canonical ligands, a large set of non-canonical ligands can activate or inhibit Notch signaling, as recently reviewed by D'Souza *et al*. [3]. Non canonical ligands include, DNER (Delta and Notch-like epidermal growth factor-related receptor) [4] F3/Contactin1, NB-3/Contactin6 [5] and Delta-like 1/2 homologue (Dlk1/2). An example of a non-canonical ligand is Dlk1/2, which is structurally similar to the Dll ligands, but lacks a DSL domain. As a consequence, Dlk1/2 fails to transactivate Notch and, therefore, acts through *cis*-inhibition by competing with canonical ligands [6]. Notch1–4 receptors are characterized by their extracellular (NECD) and intracellular domains (NICD) that respectively confer ligand recognition and signaling properties. The relative strength and specificity of receptor-ligand interactions, however, can be modulated by post-translational modifications. The extracellular epidermal growth factor (EGF) repeats of Notch receptors can be serially modified by fucosylation and glycosylation. The final glycosylation of Notch receptors mediated by Fringe proteins orients the response towards Notch ligands (Dll *versus* Jag) by promoting interactions with Dll1 and reducing interaction with Jag1 [7,8]. Alternatively, Notch can also be glycosylated by the glycosyltransferase Rumi and by at least two members of the glycosyltransferase 8 family. The impact of glycosylation, as well as the possibility to use glycosylation to modify the Notch receptors and ultimately their functions, is the subject of much investigation, as reviewed by Rampal *et al*. [9].

**Figure 1:**
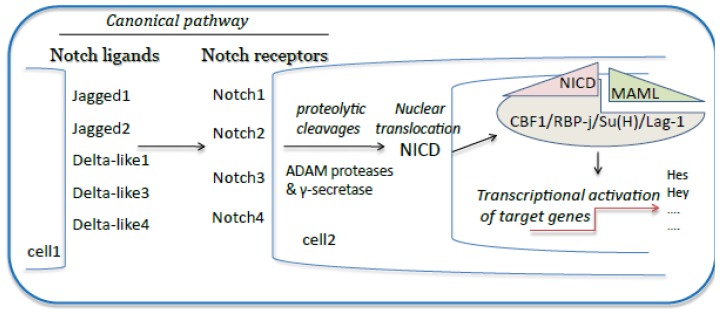
Schematic illustration of the canonical Notch signaling pathway. Notch is a heterodimeric cell-surface receptor family (Notch 1–4). Notch family members are composed of an extracellular ligand-binding domain that is non-covalently associated with a single-pass transmembrane domain. There are two distinct families of Notch ligands in mammals, known as the Jagged ligands (Jagged1 and Jagged2) and the Delta-like ligands (consisting of Dll1, Dll3 and Dll4). Dll and Jagged proteins trigger the canonical Notch signaling pathway, wherein binding of a ligand to a Notch receptor results in the cleavage of the receptor at a site in the transmembrane domain. Upon binding by either Dll or Jagged ligands, Notch undergoes proteolytic cleavages catalyzed by A disintegrin and metalloproteinase (ADAM) proteases and the γ-secretase complex, leading to the translocation of the notch intracellular domain (NICD) into the nucleus. NICD interacts with the transcriptional repressor, recombination-signal-binding protein for immunoglobulin-kJ region (RBP-J) in the CSL complex (CBF1/RBP-j/Su(H)/Lag-1). The NICD interaction with RBP-J also recruits Mastermind (MAML) protein. The new transcriptional complex of NICD-RBP-J-MAML converts RBP-J from a repressor to a transcriptional activator. The Hes (hairy and enhancer of split) proteins subsequently regulate the expression of genes involved in Notch-dependent processes, including apoptosis, proliferation or differentiation for cell-fate determination.

Activation of Notch signaling is characterized by successive proteolytic cleavages triggered by the interaction between membrane-bound Notch receptors and ligands expressed on a neighboring cell (for review, see [10]) (Figure 1). The first activating cleavage is mediated by the disintegrin and metalloproteinases (ADAM)10 or ADAM17 and follows Notch receptor/ligand interaction that mechanically expose the ADAM target site. The γ-secretase complex is responsible for the second proteolytic event that leads to the release of NICD into the cytoplasm. Following nuclear translocation, NICD forms a nuclear complex with the transcription factor, CSL (CBF1/RBP-j/Su(H)/Lag-1), and activates transcription of the downstream target genes. A molecular switch within this complex allows, finally, the expression of the main target genes of Notch signaling in mammals, including the hairy and enhancer of split (*hes*1, 5 and 7) and the Hes-related proteins (hey1 and 2 and Heyl*). Hes/hey* genes belong to a large family of transcription repressors and, therefore, regulate indirectly numerous Notch target gene expressions [2,11,12].

Intriguingly, Notch receptors and ligands are expressed on both the signal-sending cell and on the signal-receiving cell and sometimes at a roughly similar concentration. The direction of Notch signaling is given, at least partially, by the fact that ligands activate receptors on contacting cells (transactivation), but may sometimes inhibit receptors expressed in the same cell (*cis*-inhibition) [13].

A restricted distribution of Notch ligands and receptors to specific areas within cells can also contribute to signaling specificity. An alternative means to localize Notch activation is by positioning Notch ligands at cellular protrusions, such as filopodia, which leads to the activation of signaling some distance away from the signal-sending cell [14].

In the vasculature, all Notch receptors and ligands are expressed. Notch signaling has been primarily and extensively studied in blood vessel formation [15]. The crucial role of Notch in vascular homeostasis was first reported, because genetic deletion of *Notch1* or *Dll4* is lethal by inducing large defects in the vasculature, aggravated when both *Notch1* and *Notch4* are inactivated [15–19]. Notch4 and Dll4 were primarily described as endothelial-restricted molecules [16,20]. Unregulated activation of Notch4 alone also triggers vascular patterning defects [21]. Since these first reports, it is now well established that Notch pathway plays key roles in embryonic vasculogenesis. Notch signaling also plays key roles during adult life. Its implication in angiogenesis has been particularly under investigation in cancer research [22], notably by the development of Dll4 antibodies or γ-secretase inhibitors to reduce tumor growth [23].

In endothelium and vascular cells, Notch has also been reported to be an important player during inflammation. Considering the broad effects of Notch on cell biology, such implication could provide new insights about its role in the inflammatory-related vascular injury and remodeling processes involved in atherosclerosis and chronic allograft vasculopathy (CAV). This article will therefore review the current state of knowledge about the role of Notch in vascular and cardiovascular inflammatory responses.

## 2. Implication of Notch Signaling in the Pathogenesis of Cardiovascular Inflammatory Disorders

**Figure 2:**
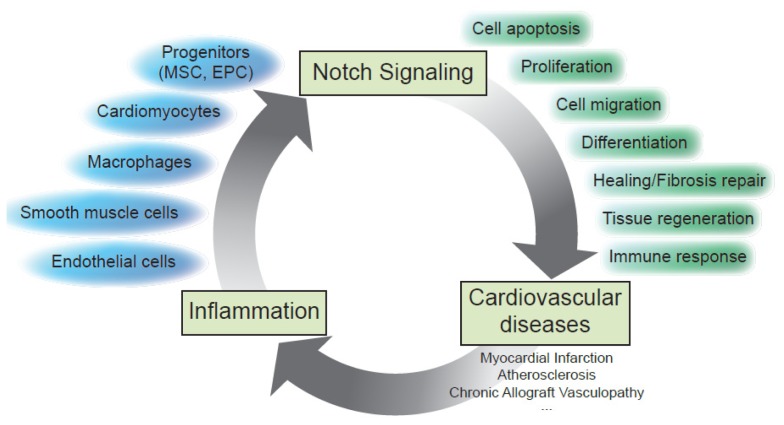
Contribution of Notch signaling in dynamic communication and cellular adaptation between immune, vascular and cardiac cells and in the control of the inflammatory reaction in damaged tissues.

Accumulative evidence supports the emerging concept that Notch signaling is central to chronic inflammatory events involved in the pathogenesis of cardiovascular diseases, and Notch may provide a new target for therapeutic approaches [24]. Notch receptors and ligands link cellular effectors and mechanisms associated with major cardiac disorders, such as myocardial infarction (MI), atherosclerosis and cardiac allograft vasculopathy (Figure 2) Myocardial infarction is the most common and clinically significant form of acute cardiac injury and results in ischemic death of a large number of cardiomyocytes. The death of ischemic cardiomyocytes elicits an inflammatory cascade that initially allows the clearing of the necrotic tissue debris within the infarct and ultimately promotes healing and repair of the damaged tissue (for review, see [25]). The Notch pathway regulates the cardiac response to stress via the Notch1 receptor [26,27]. Specifically, the receptor Notch1 and its ligand Jagged1 are the predominant members of the Notch family expressed in the adult heart. The activation of the Notch pathway in the heart in response to stress likely depends on Jagged1 expression on the surface of cardiomyocytes [28].

Notch signaling is naturally downregulated in adult compared to embryonic life. Similar to many embryonic proteins, levels of Notch1 and Hes1 decline in postnatal heart steadily after birth. Enhanced levels of Notch ligands and receptors have been reported in damaged and regenerating tissues, including heart, as well as in vessels. Augmentation of Notch activation after myocardial infarction in the adult, either by inducing cardiomyocyte-specific Notch1 transgene expression or by intramyocardial delivery of a Notch1 pseudoligand, increases survival rate, improves cardiac function and minimizes fibrosis, promoting anti-apoptotic and angiogenic mechanisms. Thus, the transient activation of endogenous Notch signaling has been observed following MI, but is insufficient to launch an effective response to cardiac damage [27]. LaFramboise *et al.* also investigated molecular changes in Notch signaling in response to myocardial infarction. The authors reported that Jag1 and Notch3 expression decreased in the infarct zone, but did not specify the cellular basis of such regulation [29]. In contrast, endothelial cells from microvessels in ischemic skeletal muscle and in myocardial tissue upregulate Dll4 expression [5]. Notch also plays key roles in the regenerative capacity of self-renewing organs. In the heart, Notch1 signaling takes place in cardiomyocytes and in mesenchymal cardiac precursors and is activated secondary to stimulated Jag1 expression on the surface of cardiomyocytes. Mice lacking *Notch1* expression in the heart demonstrated that the Notch1 pathway controls pathophysiological cardiac remodeling [30]. In the absence of Notch1, cardiac hypertrophy is exacerbated, fibrosis develops, function is altered and the mortality rate increases. An elegant model of transgenic Notch reporter (CBF1-REx4-EGFP) mice was recently used as a dynamic tool for the identification of Notch-activated progenitor cells that contribute to fibrosis repair after myocardiac injury [31].

Notch receptors also regulate the function of bone marrow (BM)-derived cells that mediate cardiac repair after myocardial injury. Interestingly, increased expression of other Notch receptors, such as Notch2 and Notch3, was observed in cardiomyocyte-restricted Notch1 knockout mice. This increase in Notch expression after infarction may be partly attributed to Notch expression in recruited BM-derived cells. Nevertheless, Notch1 signaling promotes this selective mobilization of mesenchymal stem cells (MSC) from BM-derived stem cells. The contributory role of Notch1 in BM-derived inflammatory cells, such as macrophages, in cardiac repair may be very important and needs more detailed investigation [32].

Most myocardial infarcts result from thrombotic complication of coronary atherosclerosis. Atherosclerosis is a chronic inflammatory disease resulting from interactions between lipids, macrophages/foam cells and arterial wall cells (for review, see [33]). Recent studies have identified the contribution of Notch in various mechanisms that greatly contribute to the pathogenesis of atherosclerosis. Hence, the role that the Notch pathway plays in macrophages, central to the development of inflammation and atherosclerosis, was recently reported. The first line of evidence includes the expression of multiple Notch receptors and ligands in human macrophages [34]. Dll4 and Notch3 colocalize in macrophages within atherosclerotic plaques. Dll4 binds to and activates Notch in macrophages. Notch activation leads to inflammatory gene transcription, consistent with a pro-inflammatory macrophage phenotype contributing to plaque burden, progression and thrombogenicity. Notch1 signaling is associated with macrophage activation via upregulation of expression of intercellular adhesion molecule 1 (ICAM-1) and major histocompatibility class II antigens [35]. Recent data also support the hypothesis that Notch signal activation in immune cells exacerbates atherosclerotic lesions and, in turn, that inhibition of Notch signaling by a γ-secretase inhibitor prevents or suppresses atherogenesis [36]. Consistently, blockade of Dll4-Notch signaling using neutralizing anti-Dll4 antibody attenuated the development of atherosclerosis in LDL-receptor-deficient mice [37]. Notch lateral inhibition mechanism could be extrapolated to a similar juxtacrine signaling between activated macrophages and vascular smooth muscle cell (VSMC) or valvular myofibroblasts, inducing a phenotype change in the latter. For example, a proliferative signal could be initiated in VSMC or myofibroblasts by activating Notch signaling, while having an opposite effect in endothelial cells (ECs), where Dll4 and Notch1 inhibit cell proliferation and angiogenic sprouting [38]. Endothelial cell function is also intimately involved in atherogenesis, and Notch-mediated regulation of endothelial activity can modulate the inflammatory phenotype. Little is known about the role of endothelial progenitor cells (EPC) in atherosclerosis. Kwon *et al*. have recently reported that deletion of the Notch ligand, Jagged-1, inhibits EPC-mediated angiogenesis by reducing EPC differentiation and activity [39].

Currently, cardiac allograft transplantation is the therapy of choice for patients with end-stage heart conditions. However, despite the advancements in immunosuppression, acute and chronic cardiac allograft rejection still limit the long-term graft survival cardiac allograft vasculopathy, the hallmark of chronic cardiac allograft rejection, which remains a significant cause of mortality beyond the first year of cardiac transplantation [24,40]. CAV is associated with low-grade, chronic inflammation [33]. It is thought that the inflammatory events (immune cell infiltration, cytokine and chemokine production, complement and antibody deposition, endothelial cell and smooth muscle activation) contribute to the subsequent dysfunction of the vasculature. The successive injury and repair processes are central to the intimal thickening and vascular occlusion seen in CAV. Our previous work reported that CAV correlated with an important decrease in Notch2, Notch3 and Notch4 expression in heart allografts 100 days post-transplantation. No significant change in Notch1 was observed. Downregulation of Notch4 was mainly driven by the graft endothelial cells (ECs). An extended histological analysis is still lacking to specify what cell type(s) are responsible of the overall decrease in Notch2 and Notch3 in rejected allografts [41].

Taken together, these findings support the idea that Notch signaling participates in dynamic communication and cellular adaptation between immune, vascular and cardiac cells and also in the control of the inflammatory reaction in damaged tissues (Figure 2). Thus, further understanding of the Notch pathway in the context of vascular biology likely will provide novel insights into the mechanisms of inflammation and new opportunities for rational therapeutic intervention.

## 3. Notch Signaling Is a Target of Inflammation in Vascular Cells and Inflammatory Cells

In the past decade, the Notch signaling pathway has been consistently reported to be a target of inflammatory mediators or diseases. In vasculature, all Notch family members, including ligands, are expressed at the basal level with a specific expression pattern in endothelial cells, smooth muscle cells (SMCs) and fibroblasts. Notch members are also constitutively expressed on macrophages, dendritic cells (DCs) that play a role in both vascular function and inflammation. Expression of Notch molecules correlates with a constitutive activation of the Notch pathway, as reflected by effector *hes/hey* gene expression and by CBF-1 reporter assays [17,42].

Activation by inflammatory cytokines (tumor necrosis factor (TNF), IL1β, IFNγ) triggers a change in the pattern of \Notch molecules expressed by ECs. Indeed, we recently established that, in cultured ECs, TNF and IL1β trigger a drastic decrease in Notch4 receptor and an increased Notch2 expression and activity associated with the increased presentation of Dll1 and Jag1. Interestingly, IFNγ do not modulate Notch4 expression, suggesting specific regulatory pathways and roles for the different Notch receptors in response to inflammation [43].

The Notch expression and regulation pattern can differ between ECs from various vascular beds. In human arterial ECs (HAECs), TNF triggers an increase in Dll1 ligand expression, whereas it reduces the Dll1 mRNA level in umbilical vein-derived ECs (HUVECs) (Quillard T., unpublished data). Similarly, Jag2 is induced by TNF and lipopolysaccharide (LPS) in bone marrow (BM)-derived ECs *in vitro* and *in vivo*, but this effect was not observed in HAECs [44].

In VSMCs, Notch3 has been mostly studied, because of its key implication in the cerebral autosomal dominant arteriopathy with subcortical infarcts and leukoencephalopathy (CADASIL) syndrome in humans. This pathology is triggered by Notch3 gene mutations on chromosome 19 that causes an abnormal accumulation of Notch3 NECD at the membrane of VSMCs, both in cerebral and extra-cerebral vessels [45], seen as granular osmiophilic deposits on electron microscopy. It is still unclear, though, if CADASIL-related mutations of Notch3 result in a loss or gain of function for Notch3 signaling. *In vitro*, TNF and IL1β induces a negative regulation of Notch3 expression and *hes*/*hey* effector genes [46–48]. Reduction of Notch3 signaling is thought to be crucial in the “dedifferentiation” of SMCs in vascular inflammatory disorders (see below).

Macrophages promote pathologic inflammation and angiogenesis in a large set of diseases. Recently, a specific subset of monocytes, called Tie2-expressing monocytes, with a non-inflammatory profile, has been described, supporting the concept of macrophage diversity and deciphering additional roles for macrophages in vascular development and angiogenesis [49]. Tissue macrophages, with their great mobility and flexibility and their affinity for tip-cell filopodia, are well located to help ECs on different vessel segments to establish contact [50]. By bridging tip cells from different vessel segments and embracing nascent fusion sites, macrophages act as cellular chaperones for EC fusion to increase vascular complexity and contribute to vascular morphogenesis. Thus, macrophages are indeed important para-endothelial constituents of the vascular wall [51]. Accumulation of macrophages in the vessel wall during atherogenesis could however affect Notch signaling in resident vascular cells, either by releasing inflammatory disorders or through direct intercellular communications via Notch signaling. Dll4 ligand, initially described as an endothelial-restricted molecule, is induced in macrophages by the inflammatory mediators, LPS, IL1β or oxLDL [34,52]. Moreover, macrophage activation and differentiation correlates with increased expression of Notch1, Notch2 and Notch3 receptors [34,35,53,54]. Depending on the environment, macrophages can display a spectrum of activation states ranging from classically activated, M1 inflammatory macrophages producing inflammatory mediators in response to Toll-like receptors (TLRs), to various alternatively activated M2 macrophages that are more involved in immune regulation, tissue repair, wound healing and resolution of inflammation [55]. Notch signaling appears as a regulatory pathway controlling the balance between M1 and M2 macrophage polarization. In particular, Notch signaling components, namely Dll4 ligand and the Notch1-ADAM10-γ-secretase-RBP-J axis, regulate expression of M1 genes [37,56].

Dendritic cells bridge the innate and adaptive immune responses and are major regulators of cellular immune responses. DCs are commonly observed in early vascular lesions together with macrophages and T-cells. Notch and TLR pathways interact in DCs and as a result of this interaction, DCs stimulated concomitantly with Notch and TLR ligands have a distinct cytokine profile, characterized by enhanced IL10 and IL2 and reduced IL12 expression, compared with DCs stimulated with either Notch or TLR ligands alone. This interaction between Notch and TLR signaling occurs through a non-canonical γ-secretase-independent Notch signaling pathway [57].

## 4. Notch Signaling Actively Controls Key Cellular Functions That Contribute to Inflammatory Processes

Beyond being a target of inflammation, recent studies demonstrated that Notch plays an active role in the development of inflammatory processes (see Figure 2).

### 4.1. Production of Inflammatory Factors and Immune Responses

Notch is an important regulator of immune cell differentiation and activation. Numerous studies reported its role in T CD4^+^, CD8^+^ lymphocyte, regulatory T-cell, B-cell, natural killer (NK) and dendritic cell homeostasis [58,59]. As an example, in large-vessel vasculitis, Notch blockade through Jag1-Fc or γ-secretase inhibitor treatment induces immunosuppressive effects by markedly reducing T-cell infiltration and proliferation. Th1 and Th17 responses are attenuated through the reduction of IFNγ and IL17 production in tissue, respectively [60]. Notch signaling also mediates IL10 production by T-cells via Dll4 presentation on plasmacytoid dendritic cells (pDCs) [58].

Upon atherosclerosis, monocytes/macrophages progressively accumulate in the lesions and deeply contribute to the vascular remodeling associated with plaque development [59]. In a recent study, Outtz *et al*. demonstrated that Notch1 partial deletion (Notch1^+/−^ hemizygous mice) induces a decrease in macrophage recruitment on the site of vascular injury associated with an impaired production of TNF in the wound, compared to wild-type controls. Macrophages isolated from Notch1^+/−^ subjects secrete less pro-inflammatory cytokines (TNF, IL6, IL12) and chemokines (CXCL10, CCL2) in response to IFNγ than Notch1^+/+^ derived cells [61]. Consistently, constitutive activation of Notch1 in macrophages potentiates the induction of IRF-1 (interferon regulatory factor 1), SOCS1 (suppressor of cytokine signaling-1), ICAM1 (intercellular adhesion molecule-1) and MHC-II (major histocompatibility complex, class II) by IFNγ, while reducing NO (nitric oxide) production [35]. Interestingly, Dll4 reduces the production of the IL8 chemokine in response to ischemia or TNF, while selectively inducing the expression of inflammatory genes, such as iNOS (inducible nitric oxide synthase), pentraxin 3 and Id1 [34,62]. Overall, Notch signaling in macrophages, as, to a larger extent, in the vasculitis studies, seems to favor pro-inflammatory responses by releasing more cytokines and chemokines, supposedly through Notch1 and Notch3 activation.

In contrast, in ECs, Notch4 (not present in macrophages) exhibited an anti-inflammatory function by reducing the TNF-mediated induction of vascular cell adhesion molecule-1 (VCAM-1) that plays a crucial role in the recruitment of immune cells to inflammatory sites [41].

### 4.2. Control of Vascular Cell Phenotype

Notch is a major regulator of cell phenotype. Chronic vascular inflammatory disorders that trigger vascular wall remodeling (atherosclerosis, CAV, PAH, *etc*.) are characterized notably by a progressive accumulation of VSMC/mesenchymal-like cells that exhibit low contractility and are prone to proliferate and migrate from the media to the neointima. These cells are derived from resident cells and/or circulating progenitor cells. The Notch pathway is strongly involved in cell fate control and, therefore, stands as a potentially important player in pathological remodeling processes [2]. Notch regulates differentiation and the phenotype of vascular cells.

In ECs, dysregulated activation of Notch4 by overexpressing N4ICD triggers a loss in endothelial markers (vascular endothelial [VE]-cadherin, Tie1, Tie2, platelet-endothelial cell adhesion molecule-1 and endothelial NO synthase), while inducing mesenchymal markers (alpha-smooth muscle actin, fibronectin and platelet-derived growth factor receptors) and migration toward platelet-derived growth factor-BB. Similarly, Jag1 stimulation also triggers this endothelial-to-mesenchymal transformation (EMT) [63].

Notch1 and Notch3 receptors are key regulators of VSMC differentiation [64]. Notch modulates VSMC differentiation *in vitro* through CBF-1-dependent mechanisms by controlling VSMC-restrictive genes (smooth muscle α-actin (SMA), calponin, smooth muscle myosin heavy chain (SM-MHC) and smoothelin) [65,66]. Contrasting effects have been described. The SMA promoter is a direct target of CBF1, and Notch activation in SMC is required for expression of SMA in vascular smooth muscle cells [67]. On the other hand, a more recent study showed that Hey1 and Hey2 can act as repressors of SMA expression and are also able to inhibit the initial Notch-induced SMA expression *in vitro*[68]. Hey does not block NICD/CBF-1 complex formation, although it reduces NICD/CBF-1 binding and activation of the SMA promoter [68]. Moreover, NICD stimulates VSMC differentiation by interacting with myocardin, whereas Hey2 inhibits binding of SRF (serum response factor)/myocardin to CArG elements [65]. It is, therefore, likely that fine spatial and temporal regulation of Notch activation and effector genes expression is required to control the VSMC phenotype and that Hes/Hey induction stand for a negative feedback loop to limit expression of Notch direct target genes, such as SMA.

In response to inflammatory stimuli, such as IL1β, the VSMC phenotypic transition towards an inflammatory or dedifferentiated state occurs notably through downregulation of VSMC markers and actin cytoskeleton reorganization. Activation of the Notch pathway by Dll1 or by the active form of Notch3 prevented this phenomenon, whereas Notch pathway blockade by a γ-secretase inhibitor enhanced it [47].

### 4.3. Cell Proliferation, Quiescence and Migration

In addition to regulating the cell fate of VSMCs and ECs, Notch also affects cell proliferation and migration, key cellular functions associated with vascular remodeling processes.

In primary ECs, activation of Notch4 and Notch1, through NICD transfection or through Jag1 or Dll4 presentation, inhibits proliferation [69,70]. EC growth arrest is mediated by the repression of mitogen-activated protein kinase (MAPK)/PI3K signaling and by p21^Cip1^ that prevents nuclear localization of cyclin D/cdk4 required for Rb (retinoblastoma gene product) phosphorylation and S-phase entry. Consistently, genetic or shRNA-mediated Dll4 blockade in ECs leads to increased proliferation [71,72].

The importance of Notch in vascular cell quiescence is of particular interest in the inflammatory context. Basal expression of Notch receptors and ligands in ECs and VSMCs suggest, indeed, that Notch signaling between ECs could contribute to the maintenance of endothelium quiescence. In the vasculature of the adult, it is estimated that only 0.01% of cells are actively proliferating [73,74]. As contacts between cells increase when confluence is reached, the Notch signaling pathway would therefore stand as a molecular mechano-sensor for quiescence control by inhibiting vascular cells proliferation. Noseda and others demonstrated that when ECs reach confluence, Notch signaling activity augments, while p21^Cip1^ is concomitantly downregulated [70,75]. Inhibition of Notch at confluence prevents p21^Cip1^ downregulation and induces Rb phosphorylation and proliferation.

In VSMCs, it also appears that proper regulation of Notch3 activation is required at confluence to upregulate cell cycle inhibitor p27^kip^ expression and, subsequently, block cell growth [76]. Notch signaling alters SMC growth, but in a precise manner as Notch1 and Notch3 activation, significantly increased SMC proliferation [64]. Moreover, primary VSMCs from *hey*2^−/−^ mice proliferate at a reduced rate compared with wild-type cells, whereas the over-expression of *hey1* in VSMCs leads to increased proliferation associated with reduced levels of the cyclin-dependent kinase inhibitors, p21^waf1/cip1^ (Cdkn1a) and p27^kip1^ (Cdkn1b). In this latter study, the authors demonstrated that hey2 repressor directly interacts with the p27^kip1^ promoter to block its transcription [77,78].

### 4.4. Migration, Injury Repair and Angiogenesis

Notch4 activation in human ECs from microvessels (HMECs) leads to inhibition of migration in collagen (not through fibrinogen) by increasing β1-integrin activation [79], whereas *Dll4*^−/−^ mice exhibit higher endothelial migration from the dorsal aorta to peripheral regions, which constitute the main cause of arterial lumen reduction in these embryos [71]. Notch4 inhibition by siRNA in primary HAECs limits the injury repair process in an *in vitro* wound healing assay [41]. In this study, it remains, however, unclear whether the impact of Notch4 inhibition on cell growth/survival or cell migration accounts, for the most part, in this “healing” process.

Consistently with these observations on proliferation and migration, an extensive literature depicted the role of Notch signaling in vasculogenesis and angiogenesis, especially in the perspective of cancer neoangiogenesis blockade (reviewed elsewhere [23]). Briefly, a fine tuning of Notch activation through Notch1, Notch4 and Dll4 is required for the development of mature new vessels. Blockade or over-activation of this pathway either induces higher proliferation and endothelial sprouting, leading to more immature vessels, or inhibits vessel formation, respectively. Both phenotypes are nonetheless associated with poor blood perfusion.

In VSMCs, constitutive expression of Notch1 and Notch3 intracellular domain (ICD_ results in a significant inhibition of migration. Similar results were obtained following constitutive expression of Notch1 ICD. As expected, inhibition of CBF-1 activity with RPMS-1 significantly enhances VSMC migration [64].

In more integrative models *in vivo*, *Notch*1^+/−^ (Notch1 hemizygous) and sm*Notch1*^+/−^ (SMC-restricted Notch1 hemizygosity) mice exhibit a drastic reduction of neointimal formation after carotid artery ligation compared with wild-type or control mice, whereas no significant difference was observed in Notch3^−/−^ animals. This effect in Notch1 deficient mice associates with a decreased chemotactism and proliferation and an increased apoptosis [28].

In support for a functional role of Notch pathway during the response to vascular injury, intimal hyperplasia after vascular injury is significantly decreased in *hey*2^−/−^ mice [77]. This effect correlates with reduced cellular proliferation and decreased chemotaxis and migration in response to platelet-derived growth factor (PDGF).

### 4.5. Apoptosis and Survival

Endothelial cells express many protective molecules that protect them from apoptosis (e.g., A1, A20, Bcl2, Bcl-XL, HO-1, survivin) [80]. In pathological conditions associated with endothelial dysfunction (as in chronic inflammatory disorders), ECs can undergo apoptosis. The consecutive disruption of endothelial integrity sustains inflammation and, in the worst cases, can ultimately initiate thrombotic events when underlying coagulation factors, like the von Willebrand factor, become exposed to the circulating blood [81].

In addition to its impact on cell phenotype, growth and migration, Notch also affects EC survival. The Notch4 activation level regulates EC survival. In HMECs and HUVECs, transduction of Notch4 NICD protects cells from LPS-induced apoptosis [82]. Interestingly, constitutively active CBF-1 demonstrates only partially anti-apoptotic activity by inhibiting the c-jun *N* terminal kinase (JNK)-dependent pro-apoptotic pathway. Endothelial protection can be mediated also through a CBF-1-independent mechanism that triggers the induction of Bcl-2 expression. Consistently, loss of Notch4 and Hes1 signaling by siRNA in primary HAECs elicits apoptosis [41]. In contrast, Notch2 ICD transduction in HAECs and HUVECs promotes EC apoptosis, notably by inhibiting the expression of survivin [83]. It is, therefore, likely that Notch expression pattern modulation induced by inflammatory cytokines, such as TNF, characterized by a decrease in Notch4 expression and an increase in Notch2, can act as an important event that leads to endothelial dysfunction-associated apoptosis [43].

In VSMCs, Notch1 and Notch3 activation promotes resistance to apoptosis, which is a prominent feature of the response to injury and regulates the consequent formation of the neointima [64,76,84]. Inhibition of CBF-1 activity with RPMS-1 results in a significant increase in apoptosis following serum deprivation, whereas constitutive expression of Notch3 and Notch1 ICDs protects VSMCs from death [76]. Moreover, both transient and constitutive Hey2 overexpression promotes VSMC survival in response to serum deprivation or Fas ligand, in part through the induction of Akt [78].

## 5. Crosstalks between Notch and Inflammatory Signaling Pathways

**Figure 3:**
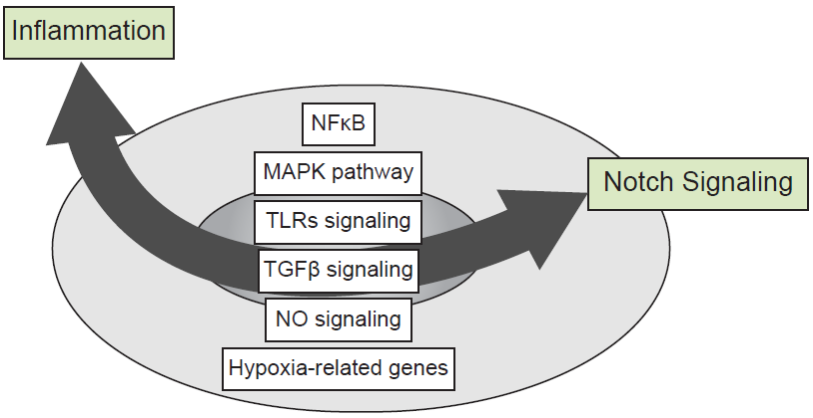
Crosstalks between Notch and inflammatory signaling pathways. The signaling network between Notch and other major inflammatory pathways is complex and includes multifaceted interactions between Notch, nuclear factor κB (NFκB), mitogen-activated protein kinase (MAPK), Toll-like receptor (TLR), transforming growth factor (TGFβ), NO and hypoxia signaling pathways.

The signaling network between Notch and other major inflammatory pathways is complex and is defined by multifaceted interactions between signaling pathways at multiple levels. Intersections have been already established with the nuclear factor κB (NFκB), mitogen-activated protein kinase (MAPK), TLR, transforming growth factor (TGFβ), NO and hypoxia pathways (Figure 3). While Notch ICD seems to be the central compound of the signaling crosstalk with other pathways, this crosstalk could, in some cases, occur independently of further CSL interaction underlining non-canonical forms of Notch signaling (for review see [85]).

### 5.1. Notch and NFκB Signaling

The NFκB signaling cascade, considered as one of the major signaling pathways leading to pro-inflammatory responses in macrophages and ECs, has been linked in several models to Notch signaling [86–88]. Notch and NFκB signaling pathways present important mutual retro-control loops. Notch1 can inhibit NFκB p50 (but not p65) by regulating molecules. A recent study showed that treatment with γ-secretase inhibitor or silencing of Notch1 decreases the translocation of NFκB p50 into the nucleus upon LPS/IFN-γ stimulation [54]. Notch1 ICD also prevents p50 fixation to DNA by direct interaction and, therefore, blocks its transcription factor function [89]. Moreover, CBF-1 repressor can interact with the IκBα promoter on a common binding site for both CBF-1 and NFκB and, therefore, favors NFκB activation by repressing IκBα expression [90]. Reciprocally, p65 sequestration in the cytosol by IκBα induces the translocation of co-repressors of Notch signaling transcriptional complex (SMRT/N-CoR) from the nucleus into the cytosol, allowing expression of Notch target genes [48]. NFκB modulates Notch signaling via both extrinsic (through Notch ligands) and intrinsic (intracellular Notch modulators) interactions [91]. Altogether, these data suggest that NFκB and Notch signaling pathways antagonize each other by various mechanisms. EC activation by TNF induces NFκB signaling through IκBα phosphorylation and degradation. NFκB p65 and p50 translocate then into the nucleus and induce NFκB target genes expression. As cited previously in this review, TNF in ECs also strongly represses Notch4 and Hes1 expression. As an illustration of the inhibitory loops between these pathways, the use of chemical inhibitors of NFκB signaling confirmed that TNF-mediated Notch impairment in ECs was dependent on NFκB, at least partially [43]. Similar findings have been reported in fibroblasts [46,48]. NFκB is also responsible for the induction of Jag1 in response to TNF in ECs [92].

### 5.2. Notch, MAPK and Innate Immunity Signaling

Interestingly, TNF-mediated induction of Notch2 receptor is not mediated by NFκB, but by the MAPK pathway. This suggests selective crosstalks between Notch and other signaling pathways and further highlights the specific functions and regulations of Notch receptors. Some reports described the communications between Notch, PI3K and MAPK pathways. Briefly, in ECs, activation of JNK induces Hes1 expression independently of Notch receptors activation. Notch1 activation reduces vascular endothelial growth factor (VEGF)-induced Akt and extracellular signal-regulated kinase (ERK)-1/2 activation [69]. In VSMCs, Notch3 signaling increases c-FLIP expression by inducing ERK1/2 phosphorylation via a CBF-1-independent mechanism [84]. Precise analysis of the direct or indirect mechanisms by which Notch integrates with such major signaling pathways remains to be defined.

Considerable evidence suggests that an innate immune defense interacts and contributes to pro-inflammatory pathways [93]. In immune cells, such as dendritic cells, Toll-like receptor signaling via myeloid differentiation factor 88 (Myd88) adaptor protein leads to the induction of the Notch ligand, Dll4, that can, in turn, activate the Notch pathway in T-cells and/or in neighboring vascular cells in inflammatory diseases [94]. Furthermore, several studies reported the contribution of Dll4 ligand in TLR9-dependent signaling in DCs, in immune responses and in venous thrombus resolution [95–97]. In macrophages, a recent study supported this interaction by showing that Notch1 and Notch2 suppressed the production of TLR4-triggered pro-inflammatory cytokines, but promoted production of anti-inflammatory cytokine IL10 through the ERK, MyD88/TRAF6 and TRIF pathways [98].

### 5.3. Notch- and Hypoxia-Mediated Signaling

A decrease in the level of oxygen induces a cellular hypoxic response. Notch signaling is implicated by several ways to the hypoxia pathway. Functional Notch signaling is required for several events of the hypoxic response, such as the control of myogenic differentiation and EMT [99,100]. One important environmental parameter in inflammatory processes that may involve Notch signaling is the pivotal role of oxygen homeostasis in the phenotypic response of ECs and VSMCs following injury or after acute myocardial infarct. HIF-1 (hypoxia-inducible factor-1) is a transcription factor that functions as a sensor to changes in available oxygen in the cellular environment, a feature implicated in many vascular diseases [101,102].

HIF-1α and Notch signaling pathways appear to be functionally integrated. The activated form of HIF-1 enhances NICD-dependent target gene expression by stabilizing its binding to the promoters, thereby providing a new mechanism by which hypoxia can regulate the responses of vascular cells [99]. Hypoxia also induces the expression of Dll4 in endothelial cells, leading to increased Notch signaling in neighboring cells [103,104]. Hypoxia also controls the expression of other Notch ligands, and Dll1 and Jag2 have been reported to be upregulated by low oxygen levels [100,105]. In pulmonary arterial hypertension, hypoxia upregulates Notch3 expression; Notch3 upregulation is a key initiating event of the disease [106]. An additional crosstalk is the interaction between NICD and the factor FIH-1 (factor inhibiting HIF-1α), that can suppress the intracellular level of HIF-α [107]. As FIH-1 binds preferentially to NICD more than HIF-1α, Notch activation indirectly enhances expression of HIF-1α-responsive genes [108]. Moreover, Notch signaling is needed to convert the hypoxic stimulus into epithelial-to-mesenchymal transition (EMT), increased motility and invasiveness. The Notch signaling pathway is an attractive candidate as a mediator for an alternative readout between hypoxia and EMT in tumor cells. Notch can upregulate *Snail-1* and induce EMT in normoxia during normal development in cardiac [109] and kidney tubular cell [110] differentiation. Although Notch implication in tumor angiogenesis in response to hypoxia has been well established, the response of Notch signaling to hypoxia in inflammatory processes still remains to be clarified.

### 5.4. Notch and NO Signaling

Nitric oxide (NO) is a major player in cardiovascular responses to inflammation. NO is generated by nitric oxide synthases (NOS), which display three isoforms: endothelial (*eNOS* or *NOS3*), inducible (*iNOS* or *NOS2*) and neuronal (*nNOS* or *NOS1*). External signals (shear stress, calcium influx or growth factors) culminate in phosphorylation events that can either activate (Ser114 and Ser1177) or inhibit (Thr485) *eNOS*[111]. Similar to hemizygous *Notch1* mutations in human [112], homozygous *eNOS* knockout mice display major cardiac anomalies [113]. Impairment of NO production by ECs notably characterizes EC dysfunction and is a key element that further contributes to cell activation and vascular remodeling [114]. A recent study demonstrates that Notch promotes endothelial-to-mesenchymal transition (EMT) by an autocrine activation of NO signaling. Notch activation in ECs induces the secretion of activin A, leading to activation of eNOS and release of NO by a PI3K/Akt-dependent mechanism [115].

Some work in macrophages and microglia (resident macrophages of the brain) also support a link between these pathways. First, Dll4 presentation to macrophages induces inducible NO synthase (iNOS) [34]. Inversely, inhibition of Notch1 in microglia cells associates with a decrease in NO production and pro-inflammatory cytokines secretion [116]. Reciprocally, stimulation of macrophages by NO reduces Notch1 signaling by blocking the interaction between NICD and CBF1 through direct NICD nitration [52]. In other biological contexts, Notch and NO seem to be interdependent. In human glioblastomas, the NO/cGMP/PKG pathway drives Notch signaling in PDGF-induced gliomas *in vitro* and induces the side population phenotype in primary glioma cell cultures [117]. Loss of NO in these tumors reduces Notch signaling *in vivo*. Similarly, in murine cholangiocytes, Notch1 expression is dependent on iNOS activity [118]. iNOS expression also facilitates NICD translocation into the nucleus and Notch target gene *hes1* expression. Inversely, the inhibition of Jag1 decreases iNOS expression in activated astrocytes [119]. Interactions between Notch and NO signaling specifically in ECs and VSMCs have not been reported yet.

### 5.5. Notch and TGFβ Signaling

Notch signaling also intersects with the transforming growth factor (TGFβ) signaling pathway. TGFβ is the prototypic member of a family of pleiotropic cytokines, including three TGFβ isoforms (TGFβ1, 2 and 3), activins and bone morphogenetic proteins (BMP) (for review, see [120]). TGFβ displays a wide range of biological effects by regulating cell proliferation, differentiation and apoptosis and modulating the immune response. TGFβ expression is elevated in response to injury. Because of its anti-inflammatory and fibrogenic properties, TGFβ may be an essential mediator for cardiac repair by mediating the transition from inflammation to fibrosis. TGFβ is also an important regulator of the EC-SMC interaction.

The TGFβ receptor is a heterodimer of TGFβ type I (termed activin linked kinase [ALK]5) and TGFβ type II receptors. ALK5 phosphorylates Smad-2 and −3, which bind to Smad4; the complex translocates into the nucleus and activates transcription of target genes. TGFβ also activates several non-canonical (SMAD-independent) signaling pathways, including: MAPK signaling cascades, RhoA-ROCK signaling and Ras signaling [121].

A direct link between Notch and TGFβ/BMP signaling is evident, given the reported interactions of Notch ICD with SMADs (SMAD3 for TGFβ; SMAD1 for BMP) [122,123]. During Notch-TGFβ cross-talk, TGFβ signaling enhances canonical Notch signaling, whereas the effect of Notch on TGFβ signaling is more multi-faceted. For example, Notch/TGFβ induction of Hey1 occurs at the expense of TGFβ-mediated induction of inhibitor of DNA binding 1 (Id1) [123]. TGFβ-mediated epithelial-to-mesenchymal transition also requires functional Notch signaling in the developing heart [109]. During endothelial-to-mesenchymal transition in cardiac cushion morphogenesis, Notch signaling inhibits SMAD1 and SMAD2 expression, but increases SMAD3 mRNA expression. SMAD3 is recruited to both SMAD and CSL binding sites to promote the downstream response [124]. The interactions between Notch NICD and SMAD appear to be receptor-specific. For example, Notch4 ICD, but not Notch1 or Notch2 ICD, interacts with phosphorylated SMAD-2 and −3 in SMCs [125]. Moreover, the latter study also supports the notion that SMADs can be recruited to the Notch transcription complex. On another hand, TGFβ downregulates the expression of Notch3 upon smooth muscle differentiation [126], indicating that aside from directly regulating smooth muscle differentiation genes, TGFβ acts through the repression of Notch3, a critical inhibitor of differentiation. Taken together, these data indicate that both TGFβ and Notch functions are context-dependent, and their subsequent effects are likely based on specific interactions with their respective signaling pathways.

## 6. Limits and Perspectives

It is clear now that inflammation alters Notch signaling in vascular cells. The main limit remains on how, in a multi-cellular complex tissue, Notch expression and activation pattern is regulated to orient the cell response to inflammation.

**Figure 4:**
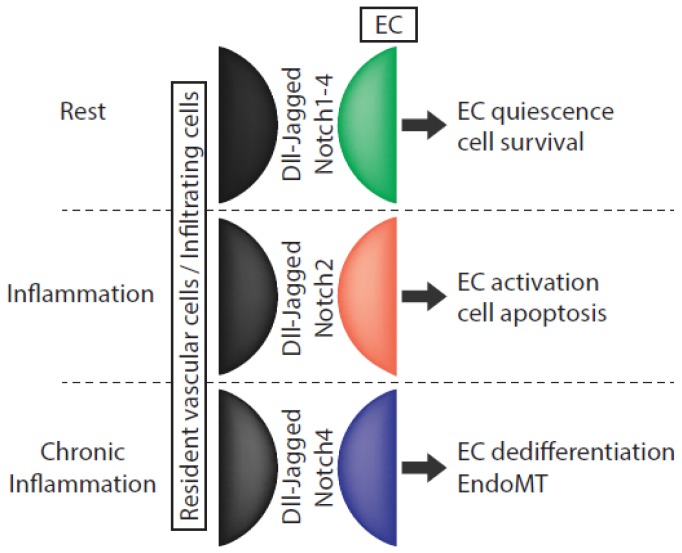
Functional impact of Notch signaling dysregulation in vascular endothelial cells (EC) upon inflammation.

An illustration of how important is to integrate Notch signaling *in situ* in a multi-cell-type communication system is that Jag1 expression on ECs is required for VSMC differentiation and development [127]. Because Jag1 can also trigger EMT [63], it also suggests that fine spatial and temporal regulation of Notch communications is crucial in a given cell type and in a heterogeneous tissue to trigger specific cell responses. These responses are likely to be dependent on what receptors/ligands combinations are involved, between resident and potentially recruited cells that can bear different sets of ligands or receptors. Parallel regulation of post-translational modifications on Notch molecules that can modify Notch receptors affinity for jag or serrate ligands are also of great importance in this matter [7,128]. Comparatively, in ECs, the net balance of the Notch signals, mediated by Notch2 and Notch4 that trigger selective responses, may orient the cell phenotype and play a critical role in the disease development (Figure 4).

Dysregulation of Notch members contributes to vascular disease development. Association of Notch3 mutations and Notch1 inactivating mutations with CADASIL syndrome and severe heart disorders, respectively, strongly support this hypothesis [45,129]. Whether CADASIL or Allagile patients (associated with Jag1 and Notch2 mutations) [130,131] also have additional defects in inflammatory responses have not been documented. Indirectly, a recent study interestingly established that CADASIL syndrome correlates with endothelial dysfunction (impairment of NO production) [132].

As our knowledge of the impact of the Notch signaling increases, the possibility arises to experimentally manipulate the Notch pathway in disease. To this aim, elegant and sophisticated studies have been performed to interfere with Notch signaling. Dll4 has been proposed as a potential target for anti-angiogenic therapy. Several studies have already reported that the Dll4 blockade inhibits tumor growth by inducing nonproductive angiogenesis manifested by an increased tumor vascular density, but a decreased tissue perfusion [133,134].

Other sophisticated strategies have been developed to interfere with specific stages of Notch signaling. For example, MAML-interfering peptides [135] or antibodies that block Notch1 receptor in an inactive state [136] have been recently developed. Unfortunately, the long-term use of such strategies might still yield unwanted side effects. In this respect, it has been shown that chronic Dll4 blockade induces vascular neoplasms [137].

The potential implications of Notch in vascular inflammatory disorders are wide, from altering cell activation, recruitment, differentiation, proliferation, migration and/or apoptosis. Nonetheless, precise analyses of its contribution in a given disease are still poorly characterized. Beyond the capabilities of Notch to modulate cell functions, much effort is now required to establish the actual role of Notch in those disorders, in a dynamic, spatial and temporal perspective. Moreover, before converting our recent knowledge into possible therapies, we also need to further elucidate the players, the functions and the regulatory events involved in the specificity and control of the Notch pathway in vascular cells.

## Abbreviations

ADAM: a disintegrin and metalloproteinase
BM: bone marrow
CADASIL: cerebral autosomal dominant arteriopathy with subcortical infarcts and leukoencephalopathy
CAV: chronic allograft vasculopathy
CBF1: C protein binding factor 1
CSL: CBF1/RBP-j/Su(H)/Lag-1
DC: dendritic cell
Dll: Delta-like
Dlk1/2: Delta-like 1/2 homologue
DNER: Delta and Notch-like epidermal growth factor-related receptor
ECs: endothelial cells
HAEC: human arterial EC
EMT: epithelial/endothelial to mesenchymal transition
EPC: endothelial progenitor cells
HUVEC: human umbilical vein EC
ICD: intracellular domain
EGF: epidermal growth factor
FIH-1: factor inhibiting
Hes: hairy and enhancer of split
Hey: Hes-related proteins
HIF-1: hypoxia-Inducible factor-1
HMEC: human microvessel EC
ICAM1: intercellular adhesion molecule-1
IFNγ: interferon gamma
IκBα: inhibitor of nuclear factor κB
IL: interleukin
iNOS: inducible nitric oxide synthase
IRF-1: interferon regulatory factor 1
Jag: Jagged
JNK: c-jun N terminal kinase
LPS: lipopolysaccharide
MALM: Mastermind-like
MAPK: mitogen-activated protein kinase
MHC-II: major histocompatibility complex, class II
MI: Myocardial infarction
NECD: Notch extracellular domain
NFκB: nuclear factor κB
NICD: Notch intracellular domain
NO: nitric oxide
oxLDL: oxidized low density lipoprotein
PDGF: platelet-derived growth factor
PI3K: phosphoinositide 3- kinase
RBP: recombination binding protein
SMA: smooth muscle α-actin
SMC: smooth muscle cells
SOCS1: suppressor of cytokine signaling-1
TGFβ: transforming growth factor
TLR: Toll like receptor
TNF: tumor necrosis factor
VCAM-1: vascular cell adhesion molecule-1
